# Advancing a Model to Account for Abnormal Spatial Relationship Perception in Bulbar Cyclotorsion

**DOI:** 10.7759/cureus.264

**Published:** 2015-04-10

**Authors:** Carlo Aleci

**Affiliations:** 1 Dept of Ophthalmology, The Gradenigo Hospital; 2 Neuro-Ophthalmology center, University of Turin

**Keywords:** cyclotropia, bulbar torsion, spatial relationship perception, aspect ratio, anisotropy, maculopapillary axis

## Abstract

In a previous study dated back to 2001, a small sample of cyclotropic patients were found to be affected by abnormal spatial relationship perception (aspect ratio judgment) with increased discrimination threshold of elliptical targets oriented along the horizontal axis. The angular amount of incyclodeviation correlated significantly with the discrimination threshold along the horizontal axis. Our group made a similar finding some years later in subjects suffering from Menière’s syndrome. In both cases, we advanced bulbar torsion to be responsible for the reduced sensibility to spatial relationship along the x-coordinate. Still, a possible explanation and a tentative model accounting for the results at that time had not been provided. This paper aims at making up for the gap, advancing a paradigm that explains the increased discrimination threshold in cyclotropic eyes as a function of the angular discrepancy between the horizontal coordinate on the retinal plane, corresponding to the maculopapillary axis (the “retinal horizon”), and the horizontal coordinate in the visual space (the “spatial horizon”). This angular discrepancy is posited to produce abnormal encoding of the spatial relationship of the target, leading to an unbalanced activation of the two antagonistic cellular pools responsible for the analysis of the aspect ratio at the cortical level. Such a model of the "dual horizon" seems to be able to account for the experimental finding described in the previous paper, providing a theoretical explanation for the defective sense of space in patients suffering from cyclotropia.

## Introduction

Based on the principles of ocular kinematics, incomitant vertical strabismus also involves, to a certain degree, a torsional component. Unlike the well-known role of the vertical deviation in inducing diplopia, the effect of such torsional component on other aspects of vision remains to be ascertained. It has been demonstrated that bulbar torsion leads to an abnormal perception of verticality, and significant correlation has been found between the former and the judgment of line orientations [1-5].

As far as we know, even if the perceptual effect of cyclotorsion reported in the literature involves the external references of the target compared to the egocentric coordinates that is the orientation of the stimuli in the environment, the perception of their *spatial relationship* or *aspect ratio* (internal references) could be affected as well.

We define spatial relationship perception (SRP) as the judgment of the spatial extent of a stimulus along its cardinal axes (vertical and horizontal). In this respect, spatial relationship (SR) is synonymous with aspect ratio. It has been suggested that this function is to be processed at the cortical level by a pool of antagonistic detectors, with each pool in charge of the horizontal or vertical cardinal coordinate [6]. (We will refer to this as V/H cortical cellular pool.) 

SRP has been investigated in previous studies in normal [6-8] and pathological conditions [9-11]. In these surveys, the visual system is found to be affected by a mild unbalance in spatial relationship perception along the cardinal axes. In particular, normal subjects show better spatial relationship discrimination along the vertical axis [7-8]; however, in certain clinical conditions like central and peripheral vertigo [11] or developmental dyslexia [10], such unbalance is shown to be more pronounced than normal, being up to twice the normative data. To our knowledge, a mechanism underlying such defective processing of spatial relations along the cardinal axes has not been theorized to date, with the exception of Sciandra [11]: she posited that ocular torsion of the eye coming from anomalous vestibular input is on the basis of defective SRP in patients suffering from vertigo. This finding prompted us more than one decade ago to provide further evidence in support of this hypothesis, assessing if torsional strabismus can affect the SRP. Indeed, we found a significant correlation between the amount of cyclodeviation and spatial relationship perception [12].

We considered such results suggestive of a link between bulbar torsion and abnormal spatial relationship perception, as we had previously suspected [11]. More specifically, incyclodeviation would cause perceptual distortion of the visual space along the horizontal axis, making the horizontal/vertical aspect ratio of the stimuli illusory higher.

Still, a possible explanation and a tentative model accounting for the results at that time had not been provided. The aim of this paper, therefore, is to make up for the gap.

## Materials and methods

Before starting the examination, the informed consent was obtained after explanation of nature and aim of the research. All applicable institutional and governmental regulations concerning the ethical use of human volunteers were followed. The research obeys the tenets of the Declaration of Helsinki.

In the above-mentioned study, nine subjects (five males, four females, median age 56 years, range 11 to 67) suffering from incomitant cyclovertical strabismus and normal, or close to normal, visual acuity were recruited. In both groups, subjective bulbar torsion had been assessed by contrast phase haploscopy [13] while SRP had been measured psychophysically according to a previously devised technique [8]. In brief, the test aims at measuring the discrimination threshold between horizontal/vertical ellipses and circles. The variable is the focal axis as well as its orientation (horizontal or vertical) while the other parameters (luminance, contrast, size) are kept constant. The eccentricity is expressed as percent interaxis ratio(IR), where the IR of a circumference is = 0:

IR (%) =100 [f(x) – f(y)] / f(m_x,y _),

where *f(x)* and *f(y)* are the two axes and *f(m_x,y _)* is the longer (the focal axis). This way, IR at threshold refers to the least eccentricity required for the stimulus to be correctly recognized as elliptical and not misperceived as a circle.

Each stimulus (average size: 300 arcmin) was displayed for 200 msec on a gray background so that its center was localized in the foveal region. The observer was required time after time to recognize the stimulus as a circle, vertical, or horizontal ellipse, according to a triple forced choice response design, and convergence to the threshold is provided by a staircase 4-2-1 procedure. The procedure was devised to estimate separately the discrimination threshold between circumferences and ellipses oriented along the cardinal axes. The test was performed dichoptically. Fusion was avoided by placing a green filter (550 nm) on the eye to be examined (cyclodeviated) and a red filter (705 nm) on the fellow eye. The red color of the target made it visible to the cyclodeviated eye, but not to the fellow eye, that in turn was made able to perceive a green small fixation cross displayed at the center of the target. This solution has been adopted since monocular examination of the cyclodeviated eye by occluding the fixating eye could normalize or reduce the torsional angle of the former.

## Results

**Figure 1:**
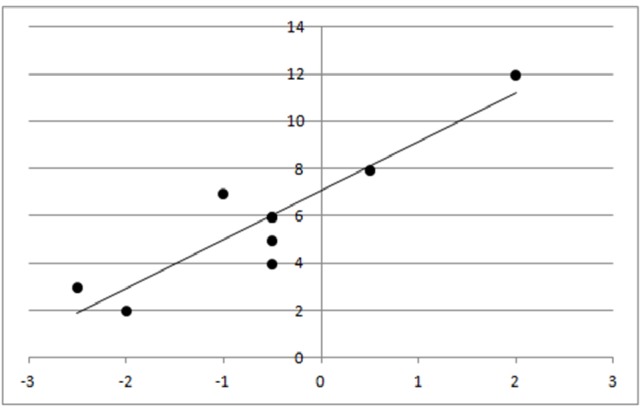
Correlation between bulbar torsion and horizontal spatial relationship perception Abscissa: bulbar torsion (deg); ordinate: IR%.

The torsion in the affected eyes ranged from -2.5 deg to +2 deg. A slight increase of SR-discrimination threshold along the horizontal axis in the paretic eyes, compared to the fellow eyes, was observed. The most interesting result, however, was a significant correlation between the amount of cyclodeviation and spatial relationship perception (r=0.91, p=0.0006) (Figure 1) [12]. 

In turn, the amount of cyclodeviation in the paretic eye did not correlate significantly with the spatial relationship perception of the fellow eye.

## Discussion

### Spatial relationship perception (SRP) and related anisotropy

Accurate SRP takes place if the ratio of the cortical processing *C* of the stimulus along the retinal cardinal coordinates, *r_y_* and* r_x_* (the proximal stimulus*, *as defined by Pizlo [14]) is proportional to the extent of the target along the spatial cardinal coordinates, *s_y_* and *s_x_* (the distal stimulus). We will call this condition SRP isotropy:

*C(r_y_ / r_x _) = s_y_ /s_x _= isotropy*                                           

It is assumed that spatial relations processing of the distal stimulus at the cortical level is based on the encoding of the proximal stimulus provided by retinal detectors: it follows that precise retinal encoding is the first basic requirement for accurate perception of the aspect ratio of the target.

In turn, inaccurate SRP would take place whenever the ratio of the cortical processing of the proximal stimulus along *r_y_* and *r_x_* is not proportional to the development of the distal stimulus along *s_y_* and *s_x_*. We will call this condition SRP anisotropy:

*C(r_y_ / r_x _) ≠ s_y_ /s_x _= anisotropy*

A main factor responsible for SRP anisotropy is the inaccurate retinal encoding of the spatial relationship of the distal stimulus (i.e., the retinal encoding is unbalanced along the y- or x-axis), so that the cortical processing of its aspect ratio, even if perfectly efficient in itself, would be biased by the retinal information. Consequently, disproportioned activation of the V/H cortical cellular pool takes place. The subjective outcome would not be veridical perception of the aspect ratio of the target.

For example, the ratio of the cortical processing of the proximal stimulus along the retinal cardinal coordinate *r_y_* and *r_x _*can be higher compared to the ratio of the extent of the distant stimulus along the spatial cardinal coordinates *s_y_* and *s_x_, *so that *C[r_y _/* *r_x _] > s_y_ /s_x_*. We will call this condition SRP-related vertical anisotropy. In the presence of such vertical anisotropy, the cortical processing of the proximal stimulus would be prevalent along the vertical axis, resulting in deformed (along the vertical) perception of the visual object. As a perceptual result of such anomalous condition, a circle would be misperceived as a vertical ellipse or a horizontal ellipse would be misperceived as less elliptical than in the visual space, or if its focal axis were small enough, as a circle. Therefore, in case of increased vertical anisotropy, the discrimination threshold of horizontal ellipses is expected to be worse. 

### A dual horizon model to account for abnormal SRP in bulbar torsion

It is worth emphasizing that the cortical cellular pool cannot directly process the spatial relationship of the distal stimulus as, in fact, such processing acts on the proximal stimulus. Therefore, the main requirement for correct spatial relationship perception would be precise retinal encoding of the spatial references of the distal stimulus. In other terms, to be accurately perceived, the SR of a visual object (distal stimulus) requires adequate encoding by the retinal detectors activated by the boundary of its retinal projection (the proximal stimulus). SRP retinal encoding is then transferred to the cortical cellular V/H pool.

Now, our model assumes that each retinal detector recruited by the target activates the two cortical pools. The strength of activation depends on the angular position of each detector relative to the horizontal coordinate in the visual space (we will refer to as “spatial horizon”).

It is assumed the retinal horizontal meridian (we will refer to as “retinal horizon”) matches the spatial horizon. So, as it occurs in orthophoric subjects, spatial relationship encoding of the retinal (i.e. proximal) stimulus accurately represents the proper spatial relationship of the external (i.e. distal) stimulus. Having accomplished this first condition, the observer will have an accurate perception of the spatial relationship of the target (provided the V/H cellular pool works well).

In this case, proper encoding of the aspect ratio of the distal stimulus is transferred via retinocortical mapping to the V/H pool. The V/H pool, in turn, will correctly process the internal spatial relations of the target based on the correct encoding of the proximal stimulus.

It follows that a stimulus more developed along the horizontal meridian, like a horizontal ellipse, is expected to activate more of the H- and less of the V-pool, so that H- and V- activation is proportional to the aspect ratio of the proximal stimulus. This will make the target perceived as a horizontal ellipse, with perceptual correspondence between the eccentricity of the distal stimulus and the percept of the observer, in confirmation of the isotropy of the visual system.

If the retinal horizon is tilted nasally as a consequence of bulbar cyclotorsion, lack of correspondence between the retinal and spatial horizon would transfer incorrect encoding of the spatial internal reference of the target to the V/H cortical pool. Such signal distortion would lead to incorrect cortical processing of the spatial relationship of the distal stimulus, making the perceived aspect ratio of the object unbalanced.

In this case, the vertical shift of the retinal detectors in relation to the spatial horizon would bias the activation of the V/H cortical pool: it follows that the activation of the V-pool would be abnormally strong compared to the H-pool. This increased V/H activation ratio would be proportional to the displacement of the detectors.

Therefore, unbalanced vertical encoding of the extent of the proximal stimulus and over activating the V-pool would make the target perceived as less extended along the horizontal than it is in the visual space. This anisotropy would account for the increased discrimination threshold along the x-axis: to be discriminated (and not misperceived as circles), horizontal ellipses must have longer focal axis (i.e., must be more “ovalized”) compared to the ordinary condition. The result, in summary, would be an illusory perception of the target as if it were less developed along the horizontal and more developed along the vertical meridian.

**Figure 2:**
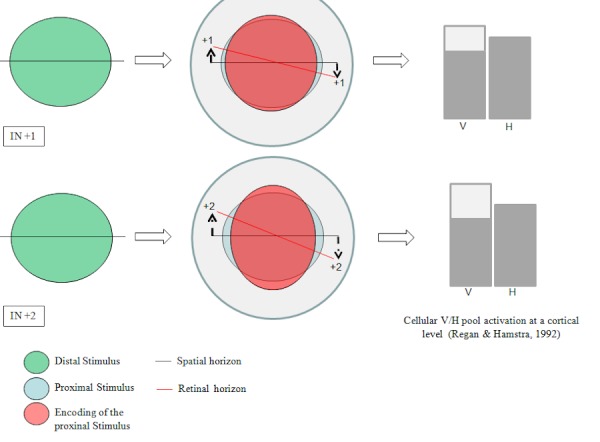
The SR encoding value of the retinal detectors depends upon their position relative to the retinal horizon (I). Defective SRP in cyclotropic eyes would derive from the occurrence of an angular difference between the retinal horizon and the spatial horizon. The difference would change the position of the retinal detectors as to the spatial horizon. At the cortical level, this discrepancy would determine over-activation of the V-cellular pool compared to the H-pool (example referred to retinal horizon tilted nasally).

To summarize, defective SRP in cyclotropic eyes would derive from the occurrence of an angular difference between the retinal horizon and the spatial horizon due to bulbar torsion. Such a tilt of the retinal horizon would induce a shift of the retinal detectors as to the spatial horizon. At the cortical level, this discrepancy would determine over-activation of the V-cellular pool compared to the H-pool (Figure 2).

The SR encoding value of the retinal detectors depends upon their position relative to the retinal horizon. Defective SRP in cyclotropic eyes would derive from the occurrence of an angular difference between the retinal horizon and the spatial horizon. The difference would change the position of the retinal detectors as to the spatial horizon. At the cortical level, this discrepancy would determine over-activation of the V-cellular pool compared to the H-pool (example referred to retinal horizon tilted nasally).

However, in which way does the positional change of the retinal detectors cause over-activation of the V-cellular pool?

We assume each retinal detector has a specific weight in encoding spatial relationship (SR-value). Each SR-value would depend on the angular distance *φ_r_*of the detector from the retinal horizon. Since retinal and spatial horizons in normal conditions (no cyclotorsion) are expected to match, the angular distance *φ_r_*of the detector from the retinal horizon corresponds to its position *φ_s_*relative to the spatial horizon.

Therefore, in the condition of no cyclotorsion, the SR-values of the retinal detectors activated by the boundary of the proximal stimulus correctly encode the aspect ratio of the target in the visual space (the distal stimulus).

**Figure 3:**
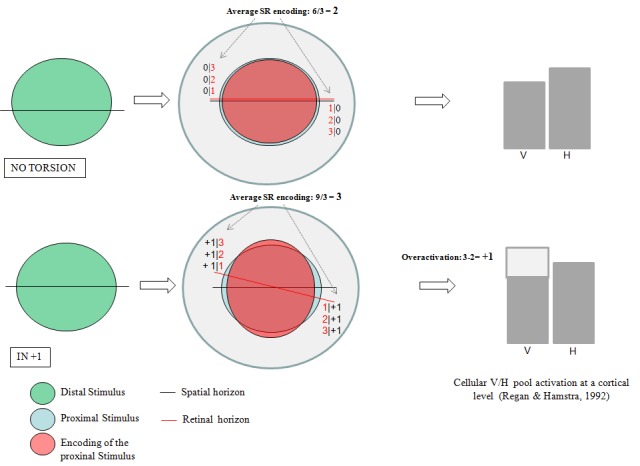
SR encoding value of the retinal detectors depends on their position relative to the retinal horizon (II). The SR encoding value of the retinal detectors depends on their position relative to the retinal horizon. In case of bulbar intorsion, the SR value increases by an amount V_l_, proportional of the angular difference between retinal and spatial horizon. The additional effect of V_l_ would lead to over-activation of the V-cortical pool, thereby to overestimation of the aspect ratio along the y-axis (increased SR threshold along the horizontal axis). Red: SR value; Black: *V_l_.* Numbers are reported only for illustrative purpose.

If a bulbar torsion *λ* occurs, *φ_r_* and *φ_s_* differ by a value *V_λ_.* In this case, the retinal detectors activated by the boundary of the proximal stimulus are shifted vertically relative to the spatial horizon by V_λ_. In their new position *φr’,* their actual response would be dictated by their proper SR-coding value depending on *φ_r_ , *plus an additional amount proportional to *V_λ_*. For nasal tilting (incyclotorsion), such a higher SR-coding value makes the encoding of the aspect ratio along the vertical preponderant, causing hyperactivation of the V-cellular cortical pool. The subjective visual outcome would be unbalanced spatial relationship perception along the vertical axis proportional to φ_r_+V_λ_, leading to increased SR discrimination threshold along the horizontal coordinate (Figure 3).

**Figure 4:**
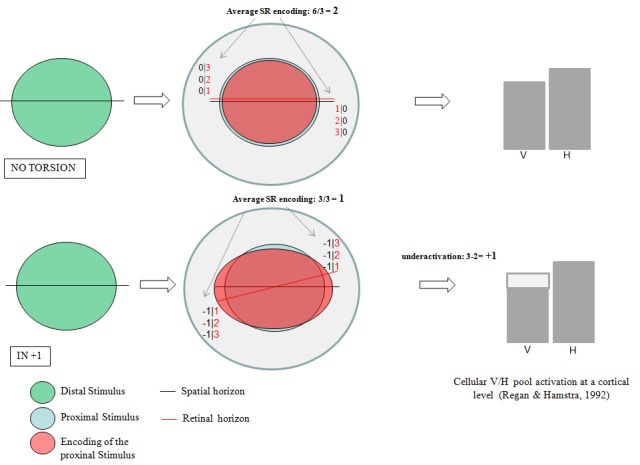
SR encoding value of the retinal detectors depends on their position relative to the retinal horizon (III). Same as Figure 3 but for bulbar excyclotorsion. Upper panels: no torsion; lower panel: extorsion. See text for explanation.

In case of excyclotorsion, the temporal tilting causes downward shift of the retinal horizon in the temporal (rather than nasal) hemiretina and upward shift of the retinal horizon in the nasal (rather than temporal) hemiretina. The reverse tilting of the retinal horizon reduces the SR-value of the retinal detectors shifted by the bulbar torsion by a value V_λ_. This reduction makes the encoding of the aspect ratio along the horizontal preponderant, causing hypoactivation of the V-cellular cortical pool (or, alternatively, hyperactivation of the H-pool). The subjective visual outcome would be unbalanced spatial relationship perception along the horizontal axis proportional to φ_r_-V_λ_; therefore, reduced SR discrimination threshold along the horizontal coordinate (conversely, increased threshold along the vertical axis is expected - see Figure 4).

The model, therefore, assumes the variation of the SR-coding value of the retinal detectors to be a function of the angular discrepancy between the retinal and the spatial horizon. Still, its vector (+V_λ_ or -V_λ_) depends on the retinal side where the shift occurs (nasal/temporal), so that:

- upward shift on the temporal side = downward shift on the nasal side = +V_λ_ in case of incyclotorsion, e.g. inferior oblique palsy.

- downward shift on the temporal side = upward shift on the nasal side = -V_λ_ in case of excyclotorsion, e.g. when trochlear palsy takes place.

To summarize, in a previous study, we advanced that the maculopapillar axis, identifiable as the retinal horizon, is tilted in subjects with Menière’s syndrome [11] as a consequence of bulbar torsion due to abnormal input coming from the vestibular system. Indeed, we ascribed the increased SRP-threshold in the sample to this angular variation.

The finding we reported in cyclotropic subjects suffering from oblique muscular hypofunction seems to confirm this hypothesis. The anisotropy referred to the aspect ratio associated with maculopapillary tilting suggests that the relation between the relative position of the retinal detectors activated by the target and the spatial coordinates in the visual space have a key role in spatial relationship encoding. According to the dual horizon model, bulbar torsion, be it a consequence of tonic abnormal vestibular input or ocular muscular deficiency, by modifying the orientation of the maculopapillary axis (the "retinal horizon”) would alter the spatial relations of the retinal detectors relative to the cardinal coordinates in the visual space (“the spatial horizon”). This change would transmit to the visual cortex a wrong input, processed by the detectors in charge of SRP analysis (the H- and V- cellular pools). Anisotropy would, therefore, stem by a “deceiving” signal provided by low-level detectors to the cortical cellular pools responsible for spatial relationship analysis, so that the more tilted the retinal horizon is, the higher would be the amount of anisotropy.

Evidently, factors other than abnormal bulbar torsion could affect spatial relationship perception. A number of studies, for example, showed that visual perception is spatially distorted in strabismic amblyopic eyes [15-24]. In particular, by using post-images, Sireteanu, et al. showed that the amblyopic eye is anisotropic [23-24]. Abnormal cortical processing of visual information could be responsible for this effect, making this case different from cyclotorsion, where the defect has been postulated to localize at a lower-level of visual processing.

## Conclusions

In conclusion, the model of the dual horizon aims at explaining the defective spatial relationship perception we have found in subjects affected by cyclotorsional strabismus as a consequence of a mismatch between spatial coordinates and retinal coordinates.

In case such a discrepancy does not take place, abnormal aspect/ratio anisotropy (if it occurs) would stem from primary cortical malfunctioning of the specific cellular systems.
